# Enzymatic characterization and structure-function relationship of two chitinases, LmChiA and LmChiB, from *Listeria monocytogenes*

**DOI:** 10.1016/j.heliyon.2020.e04252

**Published:** 2020-07-01

**Authors:** Wasinee Churklam, Ratchaneewan Aunpad

**Affiliations:** Graduate Program in Biomedical Sciences, Faculty of Allied Health Sciences, Thammasat University, Pathum Thani, Thailand

**Keywords:** Microbiology, Biotechnology, Biochemistry, Molecular biology, Infectious disease, Chitinase, *Listeria monocytogenes*, Characterization, LmChiA, LmChiB, Homology modeling, Mutagenesis

## Abstract

*Listeria monocytogenes* possesses two chitinases (LmChiA and LmChiB) belonging to glycoside hydrolase family 18 (GH18). In this study, two chitinase genes (*lmchiA* and *lmchiB*) from *L. monocytogenes* 10403S were cloned and their biochemical characteristics were studied. Using colloidal chitin as substrate, both chitinases exhibited maximum catalytic activity at pH 6–7 with optimum temperature at 50 °C. Their activities were stable over broad pH (3–10) and temperature (10–50 °C) ranges. Kinetic analysis using [4NP-(GlcNAc)_2_] as substrate indicated that LmChiB had an approximately 4-fold lower *K*_m_ and 2-fold higher *k*_cat_ than LmChiA, suggesting that the catalytic specificity and efficiency of LmChiB were greater than those of LmChiA. LmChiA and LmChiB showed the same reactivity toward oligomeric substrates and exhibited both non-processive endo-acting and processive exo-acting (chitobiosidase) activity on colloidal chitin, chitin oligosaccharides and 4-nitrophenyl substrates. Structure-based sequence alignments and homology modeling of the catalytic domains revealed that both chitinases consisted of an (α/β)_8_ TIM barrel fold with a conserved DXDXE motif. The key residues involved in the substrate hydrolysis were conserved with other bacterial chitinases. The site-directed mutagenesis of conserved Asp and Glu residues in DXDXE motif of both chitinases significantly reduced the chitinolytic activity toward colloidal chitin substrate and revealed their critical role in the catalytic mechanism. LmChiA and LmChiB might have potential in chitin waste utilization and biotechnological applications.

## 1 Introduction

Chitinases (EC.3.2.1.14) are glycosyl hydrolase (GH) enzymes that catalyze the hydrolysis of chitin, a long-chain linear polymer of *N*-acetylglucosamine (GlcNAc) through the cleavage of the β-(1,4) glycosidic bonds. Endochitinases randomly cleave glycosidic linkages of chitin chains at internal sites, generating chitooligosaccharides. Exochitinases can be divided into two subcategories: chitobiosidase and β-*N*-acetylglucosaminidase (GlcNAcases). Chitobiosidase catalyses the release of diacetylchitobiose from the non-reducing ends of the chitin chain, and β-*N*-acetylglucosaminidase cleaves the oligomers of *N*-acetylglucosamine (GlcNAc), producing GlcNAc monomer (Li and Greene, 2010).

Based on amino acid similarity of the catalytic domain, most of bacterial chitinases are classified into family 18 of glycosyl hydrolases (Matsumoto et al., 1999). Family 18 chitinases have catalytic domains of an (α/β)_8_ triosephosphate isomerase (TIM barrel) fold with a conserved DXDXE motif (Matsumoto et al., 1999; van Aalten et al., 2001). This catalytic motif contains a glutamic acid which protonates the oxygen in scissile glycosidic bonds. Binding of substrate involves a conformational change of aspartic acid which is of importance for substrate-distortion and catalysis. It was shown that the mutational effects of Asp140, Asp142 and Glu144 on chitinase B (SmChiB) from *Serratia marcescens* caused the large reduction in enzyme activity (Synstad et al., 2004; Vaaje-Kolstad et al., 2004). Apart from the catalytic domain, some family 18 chitinases also have one or more additional domains that are involved in binding to the substrate, such as chitin-binding domains and fibronectin type III domains, but some chitinases have only a catalytic domain (Hashimoto et al., 2000; Ikegami et al., 2000).

Chitinases are found in a wide variety of living organisms, including bacteria, fungi, plants, insects, crustaceans and mammals (Bhattacharya et al., 2007). In general, bacteria secrete chitinases to convert insoluble chitin in nature into GlcNAc product. In addition, chitinases of bacterial pathogens have been shown to be important, not only for nutrient acquisition and environmental survival but also for infectious processes in human and animal hosts (Larsen et al., 2011). Some chitinases can serve as virulence factors for infectious pathogens, for example, the chitinases from *Legionella pneumophila*, *Salmonella typhimurium* and *Listeria monocytogenes* (DebRoy et al., 2006; Larsen et al., 2011; Chaudhuri et al., 2010).

*L. monocytogenes* is an opportunistic, intracellular food-borne pathogen that causes human listeriosis. This bacterium is an environmental pathogen that is capable of living as a saprophyte in different habitats outside of hosts. It is ubiquitous in the soil, sewage and marine environment, and releases carbohydrate-degrading enzymes toward natural polysaccharides including chitin (Leisner et al., 2008). *L. monocytogenes* possesses two chitinases, chitinase A (LmChiA) and B (LmChiB). Both chitinases are considered important for the long-term survival of *L. monocytogenes* in minimal medium containing chitin (Paspaliari et al., 2015). It is proposed that LmChiB primarily contributes to the degradation of chitin chains, and that LmChiA serves as a virulence factor associated with enhanced pathogenicity of *L. monocytogenes*. The LmChiA secreted from *L. monocytogenes* 10403S has been shown to enhance bacterial survival *in vivo* through the suppression of the host's innate immune response, though the exact mechanism of action is unclear (Chaudhuri et al., 2010, 2013). Despite their potential importance, there are very few studies focusing on the biochemical characterization and structural elucidation of chitinases from *L. monocytogenes* 10403S. Details of the enzymatic properties of these chitinases would be useful for understanding their roles in biological process, and their future exploitation for biotechnological applications.

## 2 Materials and methods

### 2.1 Chemicals and substrates

Restriction enzymes were purchased from New England Biolabs (UK). Chitins (from shrimp shells), 4-nitrophenyl-*N*-acetyl-β-D-glucosamine [4NP-(GlcNAc)], 4-nitrophenyl *N*,*N*′-diacetyl-β-D-chitobioside [4NP-(GlcNAc)_2_], and 4-nitrophenyl *N*,*N*′,*N*″-triacetyl-β-D-chitotriose [4NP-(GlcNAc)_3_], and 4-nitrophenol standard were purchased from Sigma-Aldrich (Singapore). Colloidal chitin was prepared as described by Aunpad and Panbangred (2003). Chitin (50 g) from shrimp shell (Sigma-Aldrich) was dissolved in 300 ml of phosphoric acid and incubated at 30 °C with gentle shaking overnight. When chitin was completely digested, the mixture was then added to 1,000 ml of sterile distilled water and centrifuged at 8,000 rpm for 20 min at 4 °C. The colloidal chitin precipitate was repeatedly washed with sterile distilled water until its pH become neutral (pH = 7.0). The colloidal chitin was stored at 4 °C until further use.

### 2.2 Bacterial strains, plasmid and growth media

*L. monocytogenes* strain 10403S was kindly provided by Dr. Soraya Chaturongakul (Faculty of Microbiology, Mahidol University, Thailand). *L. monocytogenes* was grown overnight at 37 °C in TSAYE medium. The *E. coli* strains used for protein expression were *E. coli* BL21 (DE3) (for LmChiA) and Rosetta-gami B (for LmChiB). Plasmid pET23a (+) (Novagen, USA) was used as an expression vector. M9 minimal broth (1x M9 salts, 1M MgSO_4_, 1M CaCl_2_ and 20% glucose) was used for protein overexpression in *E. coli*. Ampicillin (100 μg/ml), tetracycline (3.13 μg/ml), chloramphenicol (34 μg/ml) and kanamycin (12.5 μg/ml) were added to the medium as needed.

### 2.3 Cloning of the chitinase genes from *L. monocytogenes* 10403S

Total genomic DNA from *L. monocytogenes* 10403S was extracted, using E.Z.N.A.® Bacterial DNA kit (OMEGA Bio-tek, USA), and used as a template for PCR amplification. The primer sequences were designed from the *lmchiA* and *lmchiB* genes of *L. monocytogenes* 10403S *(lmo1883* and *lmo0105)*, as the following: *lmchiA* forward primer was 5′-GGCCGCTAGCGCGACGGATGACGCTTCTGTGATGC-3′, and the reverse primer was 5′-GGCCCTCGAGTTTTGTTCCAACGATTGGACCAT-3'; *lmchiB* forward primer was 5′-GGCCGCTAGCGCTGAAAATGTACCACAGTATCG-3′, and the reverse primer was 5′-GGCCCTCGAGATTTATTAACAACCAAGGACCC-3' (the *Nhe*I and *Xho*I restriction sites are underlined). The reverse primers were designed to fuse with a 6×Histidine-tag (His-tag) at the N-terminal end of the recombinant protein. PCR reactions were performed using Phusion® DNA Polymerase (New England Biolabs, USA) under the following conditions: 98 °C for 3 min followed by 35 cycles of amplification (98 °C for 1 min, 55 °C for 1 min, 72 °C for 3 min), and a final cycle at 72 °C for 5 min. The amplified genes were digested by *Nhe*I and *Xho*I and ligated into the pET23a (+) expression vector to give the recombinant plasmids, pET-*chiA* and pET-*chiB*. Nucleotide sequences were confirmed by DNA sequencing.

### 2.4 Expression and purification of chitinase A (LmChiA) and B (LmChiB)

*E. coli* transformants were grown in M9 minimal broth at 37 °C until the optical density (OD_600_) reached 0.6, then induced with 1 mM isopropyl β-D-thiogalactopyranoside (IPTG) for 24 h at 25 °C. The cells were harvested by centrifugation and resuspended in binding buffer (20 mM Sodium phosphate, 500 mM NaCl, 40 mM imidazole, pH 7.4) before being subjected to sonication. The resulting lysate was centrifuged at 12,000 x g for 20 min at 4 °C and the filtered supernatant was applied to a Ni-Sepharose 6 fast flow column 5 ml (HisTrap FF column; GE Healthcare) equilibrated with binding buffer. The bound proteins were eluted with a linear gradient elution buffer (20 mM Sodium phosphate, 500 mM NaCl, 0–250 mM imidazole, pH 7.4). The fractions containing enzyme were then subjected to a 5 ml desalting column (HiTrap desalting column; GE Healthcare, USA) equilibrated with 20 mM sodium phosphate buffer pH 7.4 to remove imidazole. The eluted fraction was assessed for purity by 12% SDS-PAGE and staining with Coomassie brilliant blue G-250 (Bio-Rad, USA). The protein concentration in the sample was determined by the Bradford method using the Bio-Rad protein assay including a standard calibration curve constructed from bovine serum albumin (0–2 mg/ml).

### 2.5 Enzyme assay using colloidal chitin as substrate

Colorimetric measurement of chitinase was performed according to the method of Reissig et al. (1955). The assay was based on determination of the amount of reducing sugar as N-acetylglucosamine (GlcNAc) product of the enzymatic reaction. The GlcNAc from colloidal chitin hydrolysis was boiled at 100 °C in potassium tetraborate in order to generate an intermediate compound which was glucosazoline. This compound was then reacted with p-dimethylaminobenzaldehyde (pDAB) in acid condition to develop a purple color which had absorbance measured at 585 nm. Hydrolysis of colloidal chitin was determined by adding 200 μl of enzyme to 200 μl of 5% colloidal chitin. The reaction was incubated at 37 °C for 30 min and centrifuged at 10,000 rpm for 10 min at 4 °C. Supernatant (200 μl) was collected and mixed with 40 μl of 0.8 M potassium tetraborate pH 9.1. The mixture was boiled at 100 °C for 3 min, cooled, and then reacted with 1.2 ml of 1% pDAB solution at 37 °C. The absorbance from color development was measured at 585 nm. One unit of enzyme was defined as the amount of enzyme which release 1 μmol of GlcNAc end product per minute under assay conditions. A standard curve of GlcNAc was constructed over a range of concentrations (0–0.5 μmol/ml). Specific activity was calculated from the activity of purified enzyme per milligram of total protein (μmol/min/mg).

### 2.6 Effects of temperature and pH on chitinase activity and stability

The enzyme amount used in the assay was 10 μg (10 μl) and the enzyme dilution fold in the reaction (total volume of reaction = 400 μl) was about 40-fold. For optimal temperature determination, chitinase activity was assayed by incubating the enzyme with 5% colloidal chitin as a substrate in 20 mM sodium phosphate buffer (pH 7.4) at temperatures ranging from 10-80 °C. To determine optimal pH, chitinase activity was measured at different pH values in 20 mM glycine-HCl buffer (pH 3.0), 20 mM acetate buffer (pH 4.0–5.0), 20 mM phosphate buffer (pH 6.0–7.0), 20 mM Tris-HCl buffer (pH 8.0–9.0) and 20 mM glycine-NaOH (pH 10.0). To study the thermostability of enzyme, the samples were pre-incubated without substrate at various temperatures (10–80 °C) for 1 h before assaying by colorimetric method as described previously. To determine the pH stability, the enzyme solution was incubated at 4 °C for 1 h in various buffers (pH 3.0–10.0). Then to reduce the potential pH shifts caused by the enzyme solutions, the enzyme was diluted in 20 mM sodium phosphate buffer (pH 7.4) before measuring the relative activity. The assay was performed in triplicate.

### 2.7 Enzymatic assay using [4NP-(GlcNAc)_*n*_] as substrates

The product analysis of chitinase was determined using 4-nitrophenyl *N*-acetyl-*β*-D-glucosaminide [4NP-(GlcNAc)], 4-nitrophenyl *N, N′*-diacetyl-*β*-D-chitobioside [4NP-(GlcNAc)_2_] and 4-nitrophenyl *β*-D-*N, N′, N″*-triacetylchitotriose [4NP-(GlcNAc)_3_] as substrates. In a standard assay, 5 μl of enzyme (1.5 μg protein) was added to 45 μl of 300 μM substrate diluted in 20 mM sodium phosphate buffer (pH 7.4) and incubated at 37 °C for 30 min. The reaction was then terminated by adding 100 μl of 0.1 M sodium carbonate. The absorbance of the liberated 4-nitrophenolate ions at 405 nm was measured, and the absorption values were converted into concentrations through the 4-Nitrophenol standard curve (20–100 μM). One unit of enzyme was defined as the amount of enzyme that released 1 μmol of 4-nitrophenol end product per minute at 37 °C. Sodium phosphate buffer (pH 7.4) was used in the reaction instead of enzymes as a negative control. The assay was performed in triplicate.

### 2.8 Kinetic analysis

Kinetic parameters were determined using [4NP-(GlcNAc)_2_] as substrate by measuring initial activity at concentrations of 0.05–0.45 mM in triplicate. The *K*_m_ and *V*_max_ values were estimated based on Lineweaver-Burk plots. The turnover numbers (*k*_cat_) were calculated by dividing *V*_max_ by the enzyme concentration in the reaction mixture. The molecular mass was used to calculate the enzyme concentration (μM).

### 2.9 Analysis of products from oligomeric substrates

The enzymatic activities toward chito-oligosaccharides (GlcNAc)_*n*_ (*n* = 3–6) (Megazyme, Ireland) were measured. The reactions were assayed by mixing enzyme (0.1 μg) with 1 mM of chito-oligosaccharides substrates. The mixture was incubated at 37 °C for 10 and 30 min, and the reaction was then boiled for 10 min at 95 °C to inactivate the enzyme. The reaction mixture was centrifuged at 4 °C, 12,000 rpm for 15 min and the supernatant was then collected to analyze the degradation products. Products were detected by the Waters Alliance 2695 Separations Module HPLC system (SelectScience®, UK) using a carbohydrate analysis column (3.9 × 300 mm) (Waters, Ireland). A 50 μl of sample was injected on the column and the chitin fragments were eluted at a flow rate of 1 ml/min with 75% acetonitrile water. The chito-oligosaccharides were monitored by measuring absorbance at 215 nm. Monomer, dimer, trimer, tetramer, pentamer, and hexamer (Megazyme, Ireland) were used as standards.

### 2.10 Homology-based modeling

Homologous structure searches for LmChiA and LmChiB catalytic domains were performed using the BLASTP search tool against the PDB database to identify the template sequences which shared the highest sequence identity. The templates with over 30% sequence identity to target proteins were selected for model building to predict a structural model with an accuracy equivalent to a crystallographic structure (Xiang, 2006). The 3D-models were generated by automated protein homology-based molecular modeling using SWISS-MODEL. The loop structures of templates were applied to target amino acid sequences for loop modeling and the side chains were then computed and generated using a backbone-dependent rotamer library (Shapovalov2011). The structural model refinement and energy minimization were performed using WinCoot program version 0.8.6 (Emsley et al., 2010). The 3D-models were visualized in PyMOL 2.0 and further validated using the PROCHECK program (Laskowski et al., 1993) and a Qualitative Model Energy ANalysis (QMEAN) server (Benkert et al., 2007).

### 2.11 Site-directed mutagenesis

Site-directed mutagenesis was performed to verify the significance of conserved amino acid residues in DXDXE motif of LmChiA and LmChiB. PCR based site-directed mutagenesis method was conducted by Bionics (Seoul, Republic of Korea). The mutagenic primers contained the desired mutation are listed in Table 1. Amplification reactions were performed with a condition consisting of predenaturation at 95 °C for 30 s, followed by 18 cycles of denaturation at 95 °C for 30 s, annealing at 58 °C for 30 s and extension at 72 °C for 15 min. The mutagenized PCR products were digested by restriction enzymes (*Nhe*I and *Xho*I) and ligated to pET23a (+). The recombinant plasmids and mutated sequences were confirmed by restriction mapping and nucleotide sequencing, respectively. Each mutant plasmid was transformed into *E. coli* competent cell and the resulting strain was induced with 1 mM IPTG at 25 °C for 24 h. All clones were successfully expressed and the cells were harvested by centrifugation and resuspended in 20 mM Sodium phosphate, pH 7.4 before being subjected to sonication. The resulting lysate was centrifuged at 12,000 x g for 20 min at 4 °C and the supernatant (10 μg protein) containing chitinase was collected for chitinase activity determination toward colloidal chitin substrate as previously described. Each enzymatic assay was performed for triplicate.

**Table 1:** Primers used for site-directed mutagenesis. The underline sequences were targeted for alanine substitution.

Mutant	DND template	Primer name	Primer sequence
D159A	ChiA wild type	ChiA-D159A_S	5′-ATGGCTTTGATGGATTAGCCATCGACTTAGAACAAAG-3′
		ChiA-D159A_AS	5′-CTTTGTTCTAAGTCGATGGCTAATCCATCAAAGCCAT-3′
D161A	ChiA wild type	ChiA-D161A_S	5′-TTGATGGATTAGACATCGCCTTAGAACAAAGTGCGAT-3′
		ChiA-D161A_AS	5′-ATCGCACTTTGTTCTAAGGCGATGTCTAATCCATCAA-3′
E163A	ChiA wild type	ChiA-D163A_S	5′-GATTAGACATCGACTTAGCCCAAAGTGCGATTACCGC-3′
		ChiA-D163A_AS	5′-GCGGTAATCGCACTTTGGGCTAAGTCGATGTCTAATC-3′
D168A	ChiB wild type	ChiB-D168A_S	5′-CCAATATGGATTTTGTTGCCTTGGACTGGGAATATCC-3′
		ChiB-D168A_AS	5′-GGATATTCCCAGTCCAAGGCAACAAAATCCATATTGG-3′
D170A	ChiB wild type	ChiB-D170A_S	5′-TGGATTTTGTTGATTTGGCCTGGGAATATCCTGCTTC-3′
		ChiB-D170A_AS	5′-GAAGCAGGATATTCCCAGGCCAAATCAACAAAATCCA-3′
E172A	ChiB wild type	ChiB-D172A_S	5′-TTGTTGATTTGGACTGGGCCTATCCTGCTTCGGTACG-3′
		ChiB-D172A_AS	5′-CGTACCGAAGCAGGATAGGCCCAGTCCAAATCAACAA-3′

## 3 Results

### 3.1 Cloning and sequence analysis

The *lmchiA* and *lmchiB* genes, omitting the signal peptide sequences, were successfully cloned into the pET23a (+). The full-length LmChiA comprises a single 283-aa catalytic domain. In contrast, LmChiB was a multi-domain chitinase containing a 398-aa catalytic domain (CatD), a 70-aa fibronectin type III-like domain (FnIIID) and a C-terminal 40-aa chitin-binding domain (ChBD). According to the CAZy database, which describes the families of structurally-related catalytic and carbohydrate-binding modules (or functional domains) of enzymes that degrade, modify, or create glycosidic bonds, LmChiA and LmChiB of *L. monocytogenes* 10403S were classified into the glycosyl hydrolase family 18 (GH18).

**Figure 1:**
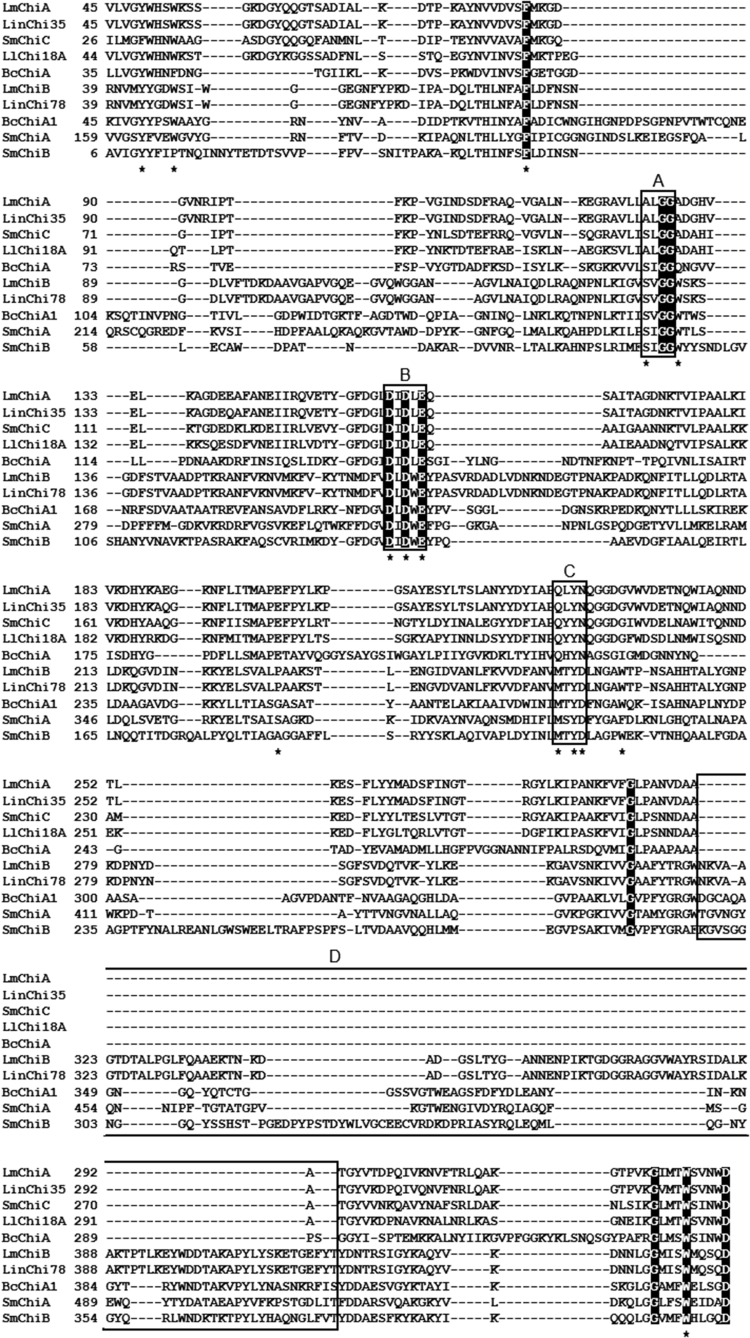
Multiple sequence alignments of the catalytic domains of LmChiA and LmChiB with those of well-characterized GH18 chitinases. Reference protein sequences include SmChiC from *S. marcescens* (PDB code: 4AXN), LlChi18A from *L. lactis* (PDB code: 3IAN), ChiNCTU2 from *Bacillus cereus* (PDB code: 3N11), BcChiA1 from *B. circulans* (PDB code: 1ITX), SmChiA from *S. marcescens* (PDB code: 1EDQ) SmChiB from *S. marcescens* (PDB code: 1E15), and LinChi35 (Genbank Accession Number: LC092876) and LinChi78 (Genbank Accession Number: LC092875) from *L. innocua*. The positions with conserved residues are shaded in black. The signature conserved motifs of GH18 chitinases, the SXGG, DXDXE and QXYN motifs, are indicated in boxes A, B and C, respectively. The (α + β)-fold insertion domain (CID) is indicated in box D. The key amino acid residues that play roles in substrate binding and catalysis in well-studied GH18 chitinases are indicated by asterisks.

**Figure 2:**
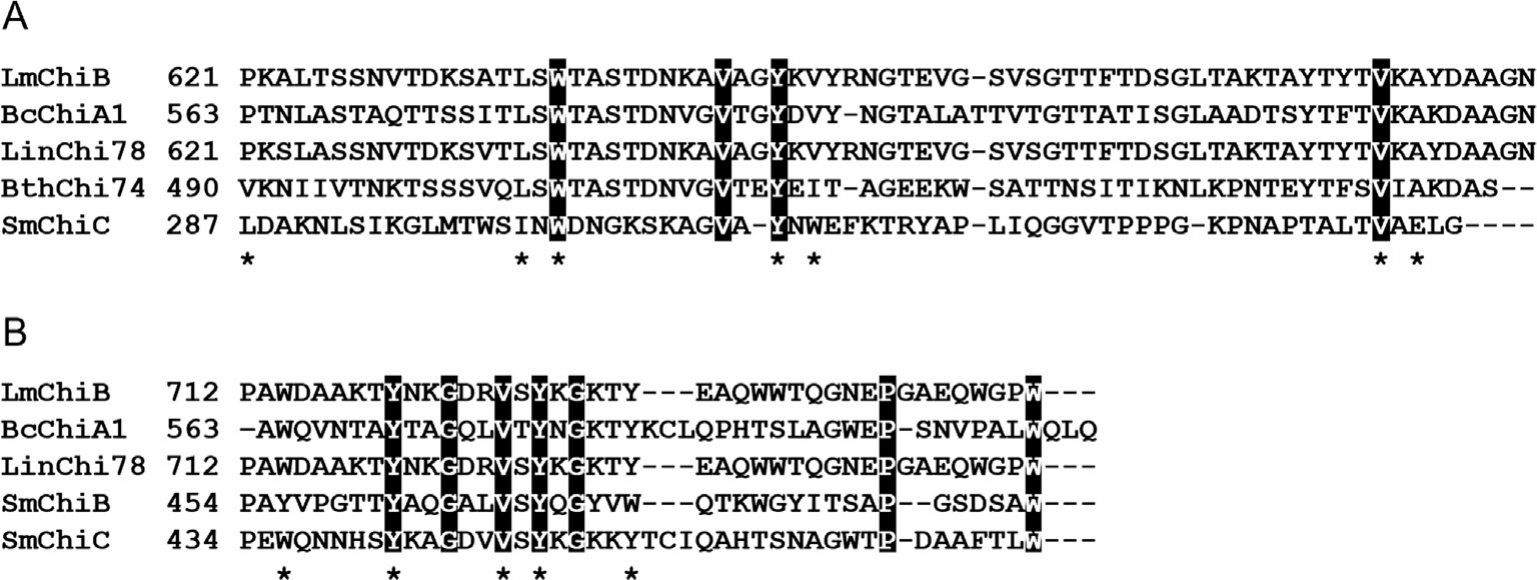
Multiple sequence alignments of the FnIIID (A) and ChBD (B) of LmChiB with those of well-characterized GH18 chitinases. Reference protein sequences for FnIIIDs include BcChiA1 from *B. circulans* (PDB code: 1K85), LinChi78 from *Listeria innocua*, BthChi74 from *Bacillus thuringiensis* and SmChiC from *S. marcescens*. Reference protein sequences for ChBDs include BcChiA1 from *B. circulans* (PDB code: 1ED7), LinChi78 from *L. innocua*, SmChiB and SmChiC from *S. marcescens*. The highly conserved amino acid residues are highlighted in black. The key amino acid residues of FnIIID and ChBD in BcChiA1 from *B. circulans* involved in substrate binding and catalysis are indicated by asterisks.

Multiple sequence alignments of the LmChiA and LmChiB catalytic domains with other related and well-characterized chitinases (Figure 1) revealed that LinChi35 is most closely related to LmChiA (97% sequence identity), whereas LmChiB is highly similar to LinChi78 with 99% sequence identity. The signature structural motifs involved in the catalytic reactions of GH18 chitinases are well conserved in both LmChiA (^159^DXDXE^163^ and ^228^QXYN^231^) and LmChiB (^168^DXDXE^172^). The DXDXE motif serves as a catalytic center of GH18 chitinases whereas QXYN motif is involved in the substrate hydrolysis (Synstad et al., 2004). In addition, LmChiB also contained the ^48^SXGG^51^ motif that is essential for the binding of substrate. The SXGG motif is followed by an important tryptophan residue which is required for the efficient to hydrolysis of insoluble chitins. This tryphtophan was also found in exochitinases (SmChiA and SmChiB) from *S. marcescens* (Figure 2A) (Horn et al., 2006). The fibronectin type III-like domain of LmChiB was related to that of *Bacillus circulans* chitinase (BcChiA1) (Figure 2A). The conserved region included Pro621, Leu636, Trp638, Try650, Val652, Val682, and Ala684 (corresponding to Pro563, Leu578, Trp580, Tyr592, Val594, Val624, and Ala626 of FnIIID in BcChiA1) that are conserved in other bacterial FnIIIDs and involved in forming a hydrophobic core of FnIIID in BcChiA1 (Jee et al., 2002). The chitin-binding domain of LmChiB was a member of family 5/12 carbohydrate-binding module based on its CAZy classification. The six aromatic and hydrophobic residues (Trp714, Try720, Try728, Tyr733, Trp752, and Val726) were conserved with ChBD of BcChiA1 (Trp656, Tyr662, Tyr670, Tyr675, Trp696, and Val668) (Ikegami et al., 2000) (Figure 2B).

### 3.2 Expression and purification of LmChiA and LmChiB

**Figure 3:**
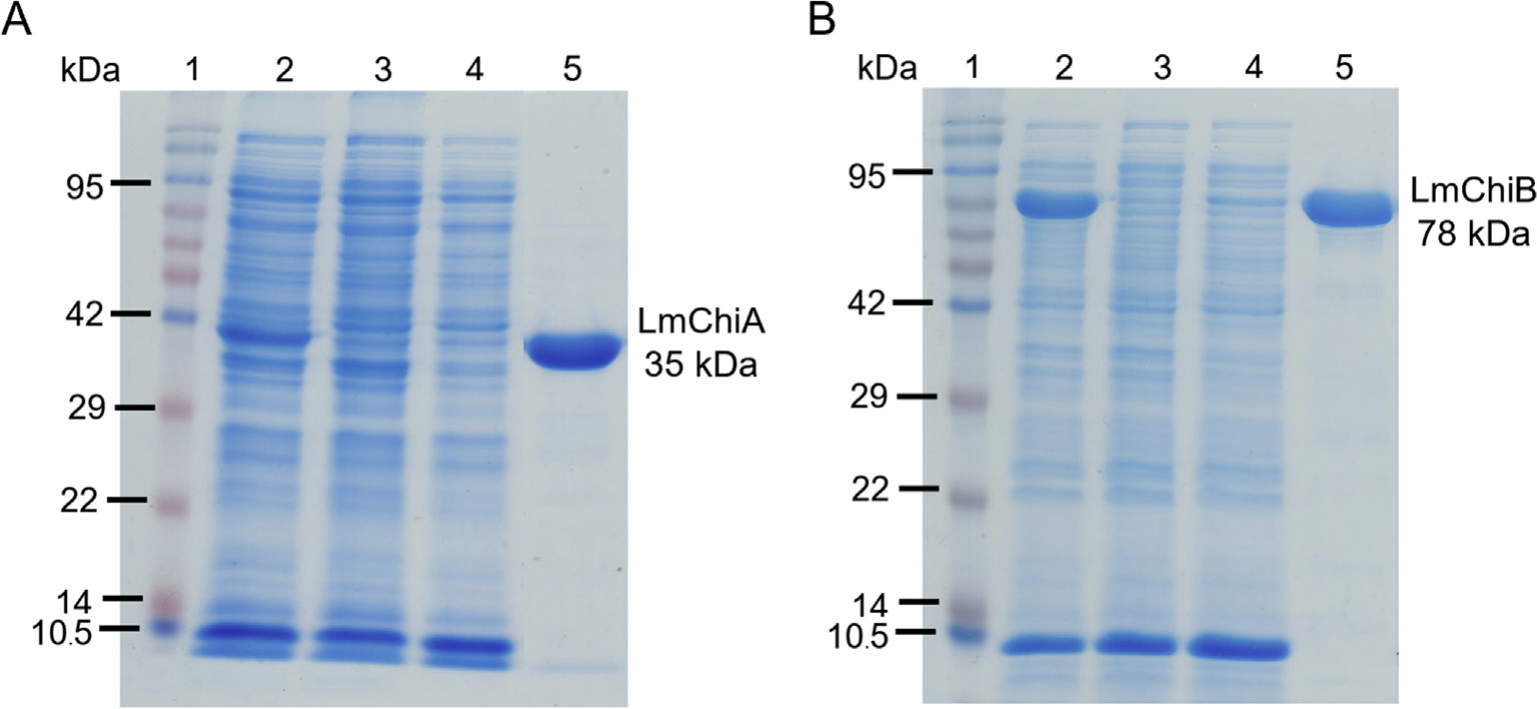
SDS-PAGE analysis of LmChiA (A) and LmChiB (B) during purification steps. Lane 1, protein marker (Chromatin Prestained Protein Ladder, Vivantis, Malaysia); lane 2, crude cell extract; lane 3–4, washing fractions after using Ni sepharose column; lane 5, purified LmChiA and LmChiB. The numbers in the margin indicate the molecular weights (kDa) of the proteins in the protein marker. A full, unmodified version of this figure is available as Supplementary File 1.

Soluble LmChiA and LmChiB were overexpressed at a relatively low temperature (25 °C) in *E. coli* BL21 (DE3) and Rosetta-gami B, respectively. SDS-PAGE analysis revealed single protein band of LmChiA and LmChiB with approximate molecular masses of 35 and 78 kDa, respectively, consistent with their theoretical molecular masses (Figure 3). The degree of enzyme purity in each purification step was also determined based on their specific activities against colloidal chitin substrate. As shown in Table 2, 15.4-fold and 2.4-fold purifications of LmChiA and LmChiB were obtained from Ni-NTA column chromatography. The recovery yields of protein after purification of LmChiA and LmChiB were 70.8% and 37.7%, respectively, compared to before purification (100%), suggesting that the total activity of LmChiA in protein mixture could be easily recovered after purification.

**Table 2:** Purification procedure of the recombinant proteins.

Protein	Purification steps	Total activity (U)	Total proteins (mg)	Specific activity (U/mg)	Purification (fold)	Recovery yield (%)
LmChiA	Cell free supernatant Ni-NTA chromatography	1209.6 856.8	155.1 7.1	7.8 120.2	1.0 15.4	100.0 70.8
LmChiB	Cell free supernatant Ni-NTA chromatography	9182.2 3458.6	102.2 15.9	89.8 217.8	1.0 2.4	100.0 37.7

Assays were performed at 37 °C in sodium phosphate buffer pH 7.4 using 5% colloidal chitin as substrate.

### 3.3 Effects of temperature and pH on chitinase activity and stability

**Figure 4:**
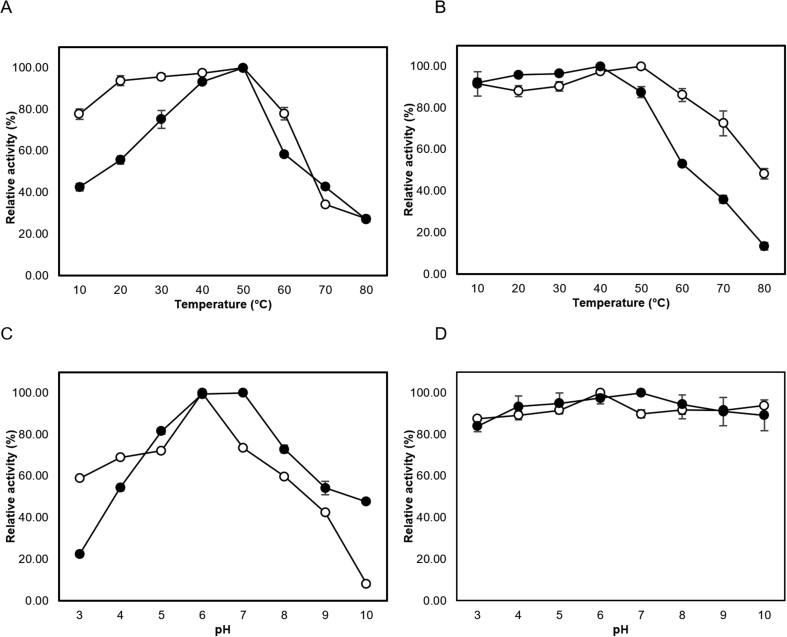
Effect of temperature and pH on the activity and stability of ChiA (open circle) and ChiB (closed circle). (A) Optimum temperature determination, (B) Thermostability determination, (C) Optimum pH determination, (D) pH stability determination. Relative activity was expressed as a percentage based on the maximum activity at 50 °C. The relative activity (%) is represented by the average values with error bars. Experiments were performed in triplicate.

Using colloidal chitin as a substrate, the maximum activity of both LmChiA and LmChiB were obtained at 50 °C (Figure 4A). The chitinase enzymes were relatively stable for 1 h in the temperature range of 10–50 °C with relative activities more than 80%. Thermostability of both enzymes was progressively decreased as temperature increased from 60 to 80 °C. LmChiB was less stable than LmChiA and almost completely inactivated (13% of activity remained) at 80 °C for 1 h (Figure 4B). LmChiA exhibited its maximal activity at pH 6.0, whereas the optimum pH of ChiB was pH 7.0 (Figure 4C). Both LmChiA and LmChiB maintained over 80% of their initial activity after 1 h of incubation at pH values ranging from 3.0-10.0 indicating their broad pH stability (Figure 4D).

### 3.4 Substrate specificity and kinetic analysis

Colloidal chitin substrate was used to screen chitinase activity of both chitinases. As shown in Table 3, the specific activities of LmChiA and LmChiB toward colloidal chitin were determined to be 4.23 and 17.06 U/μmol, respectively. LmChiB showed higher chitinolytic activity than LmChiA, possibly associated with the structural domains of chitinase. LmChiB contains a chitin binding domain that helps to increase the substrate affinity and catalytic efficiency of the enzyme (Yan and Fong, 2018), whereas LmChiA lacks this domain. 4-nitrophenyl substrates including [4NP-(GlcNAc)], [4NP-(GlcNAc)_2_] and [4NP-(GlcNAc)_3_] were assayed to detect GlcNAcase, chitobiosidase and endochitinase activity, respectively. Both chitinases could hydrolyze [4NP-(GlcNAc)_2_] and [4NP-(GlcNAc)_3_]. LmChiA exhibited higher chitinase activity toward [4NP-(GlcNAc)_3_], whereas LmChiB was more effective against [4NP-(GlcNAc)_2_]. No activity was detected toward [4NP-(GlcNAc)] substrate for both LmChiA and LmChiB. From the results, LmChiA and LmChiB exhibited both endo-acting and exo-acting (chitobiosidase activity) on 4-nitrophenyl substrates. The kinetic parameters of each enzyme were determined using [4NP-(GlcNAc)_2_] as a substrate. The *k*_cat_ value of LmChiB was 2-fold higher than that of LmChiA, suggesting that the catalytic efficiency of LmChiB was greater than that of LmChiA for [4NP-(GlcNAc)_2_] hydrolysis (Table 4). The *k*_cat_ indicates the number of substrate molecules converted into product by one molecule of enzyme active site per unit time (when enzyme is fully saturated with substrate). The *k*_cat_ of LmChiB was expected to be higher than that of LmChiA due to its additional chitin-binding domain that facilitates substrate hydrolysis (Yan and Fong, 2018).

**Table 3:** Specific activity against colloidal chitin and artificial substrates of LmChiA and LmChiB.

Substrate	Specific activity (U/μmol)	
	LmChiA	LmChiB
Colloidal chitin∗	4.85 ± 0.57	17.53 ± 0.58
4-nitrophenyl-*N*-acetyl-β-D-glucosamine [4NP-(GlcNAc)]∗∗	0	0
4-nitrophenyl *N*,*N*′-diacetyl-β-D-chitobioside [4NP-(GlcNAc)_2_]∗∗	20.02 ± 0.86	20.64 ± 1.13
4-nitrophenyl *N*,*N*′,*N*″-triacetyl-β-D-chitotriose [4NP-(GlcNAc)_3_]∗∗	22.75 ± 0.53	12.10 ± 0.44

∗ For assays using colloidal chitin substrate (5% final concentration), one unit was defined as the amount of enzyme which released 1 μmol of GlcNAc/mg/min under assay condition.

∗∗ For assays using artificial substrates (0.3 mM final concentration), one unit was defined as the amount of enzyme which released 1 μmol of 4-nitrophenol/mg/min under assay condition.

**Table 4:** Kinetic parameters of two GH18 chitinases for 4NP-(GlcNAc)_2_ substrate.

Enzymes	*K*_m_ (μM)	*k*_cat_ (s^−1^)	*k*_cat_/*K*_m_ (s^−1^ μM^−1^)
LmChiA	1622 ± 13	33.57 ± 1.0	0.02
LmChiB	375 ± 27	72.11 ± 1.4	0.19

### 3.5 Detection of hydrolysis products from oligomeric substrates

**Figure 5:**
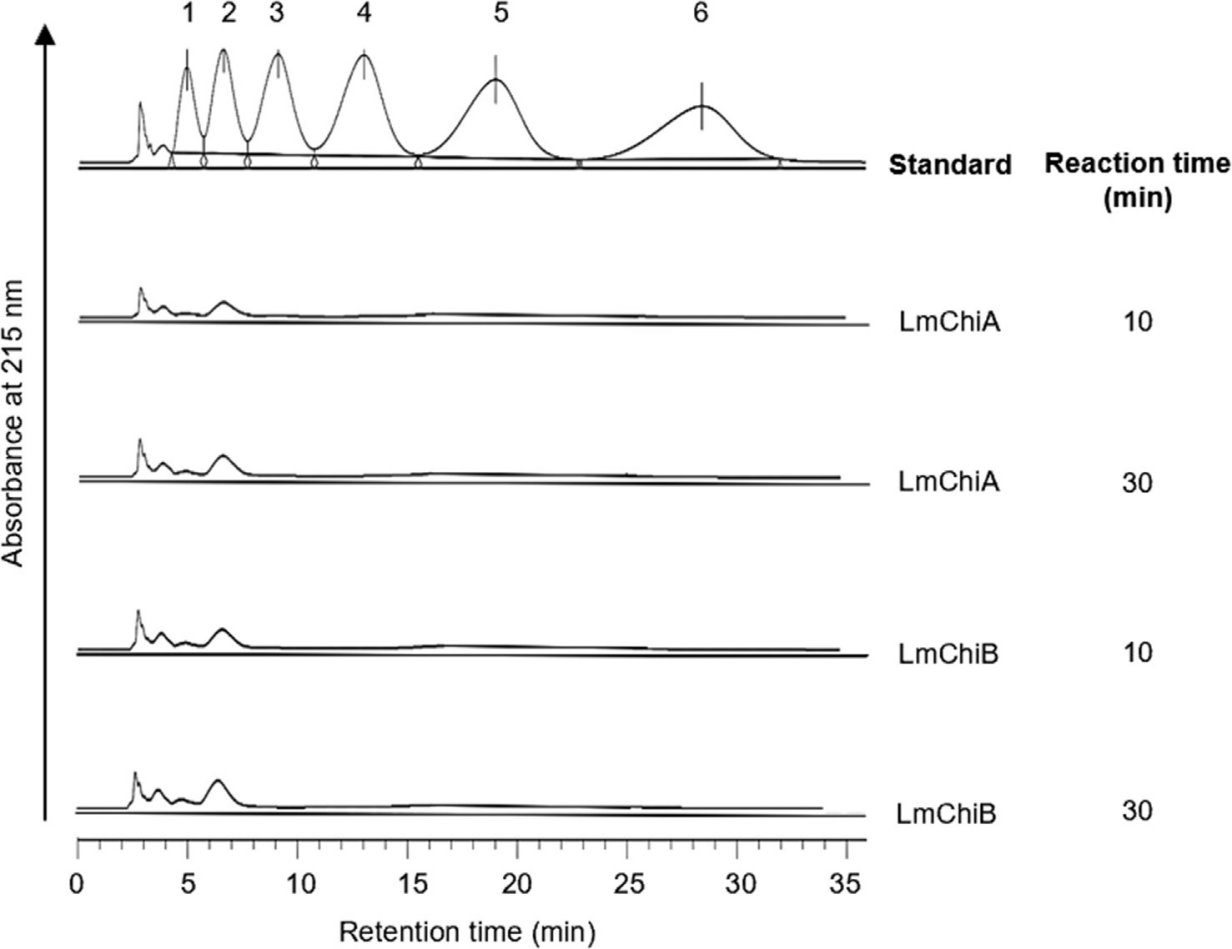
Degradation products from colloidal chitin digestion by LmChiA (A) and LmChiB (B) analyzed by HPLC. Chito-oligosaccharides were used as standards.

**Figure 6:**
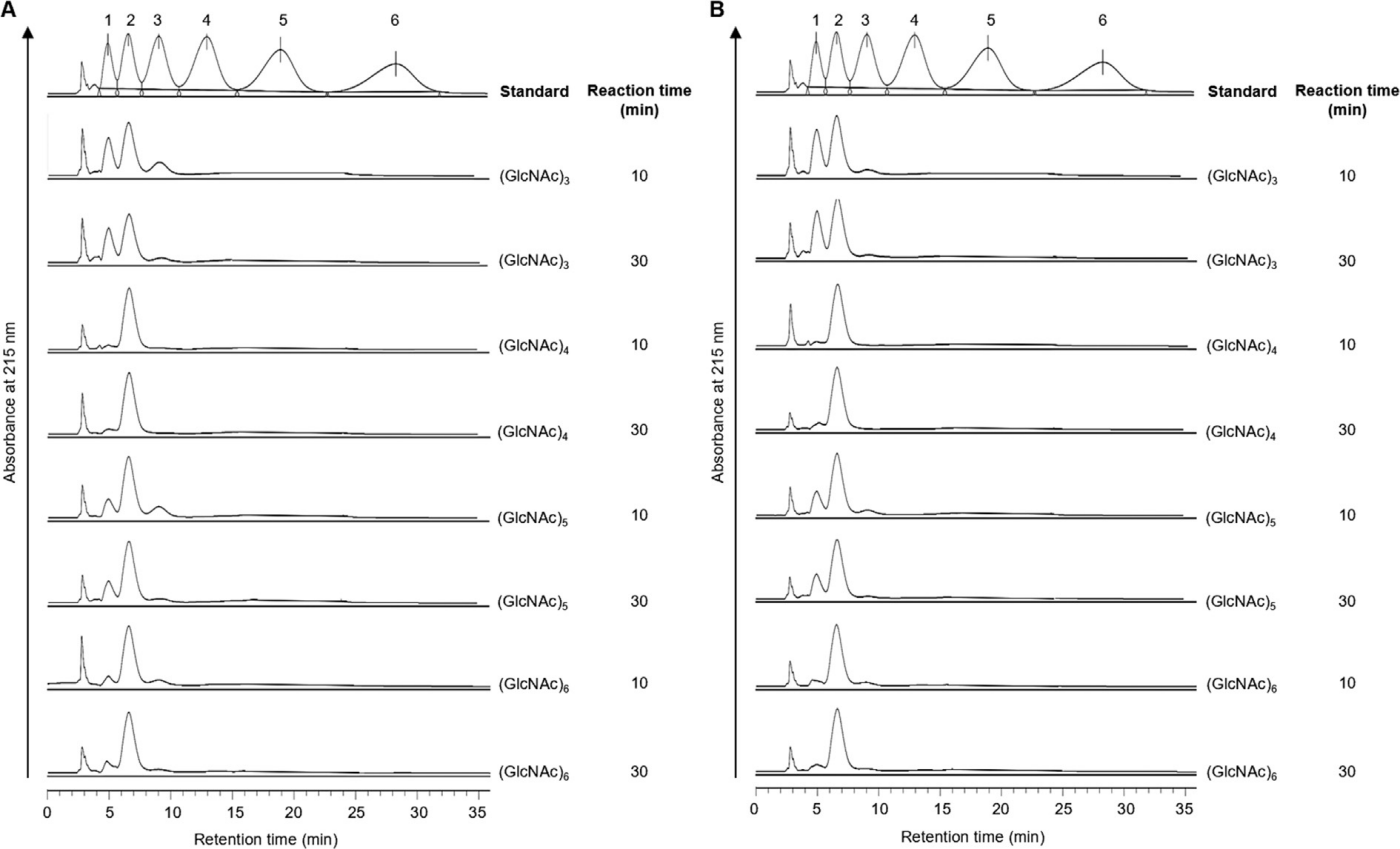
HPLC analysis of (GlcNAc)_*n*_ hydrolysis products by LmChiA (A) and LmChiB (B). The reactions were performed at 37 °C for 10 and 30 min. Chito-oligosaccharides were used as standards.

To investigate the hydrolysis mechanisms of LmChiA and LmChiB, colloidal chitin and chito-oligosaccharides (GlcNAc)_*n*_ (*n* = 3–6) were used as substrates. Time course of product distributions at 10 and 30 min of both chitinases were analyzed. The major product obtained by both chitinases after 10 and 30 min of reaction with colloidal chitin (Figure 5) and chito-oliogosaccharides (Figure 6) was dimer (GlcNAc)_2_, with a smaller amount of monomer (GlcNAc) indicating their exochitinase (chitobiosidase) activity. Under the substrate concentration tested (1 mM), both chitinases efficiently hydrolyzed all oligomeric substrates to dimer (GlcNAc)_2_ and there was no tetramer detected even at 10 min with (GlcNAc)_6_ substrate indicating processive activity. LmChiA and LmChiB produced trimer (GlcNAc)_3_ from (GlcNAc)_6_ substrate in a time-dependent manner but showed very weak hydrolytic activity suggesting non-processive endochitinolytic activity. Both chitinases showed the same reactivity toward all oligomeric substrates, and the hydrolysis products did not change much with the duration of incubation.

### 3.6 Homology-based modeling of LmChiA and LmChiB catalytic domains

**Figure 7:**
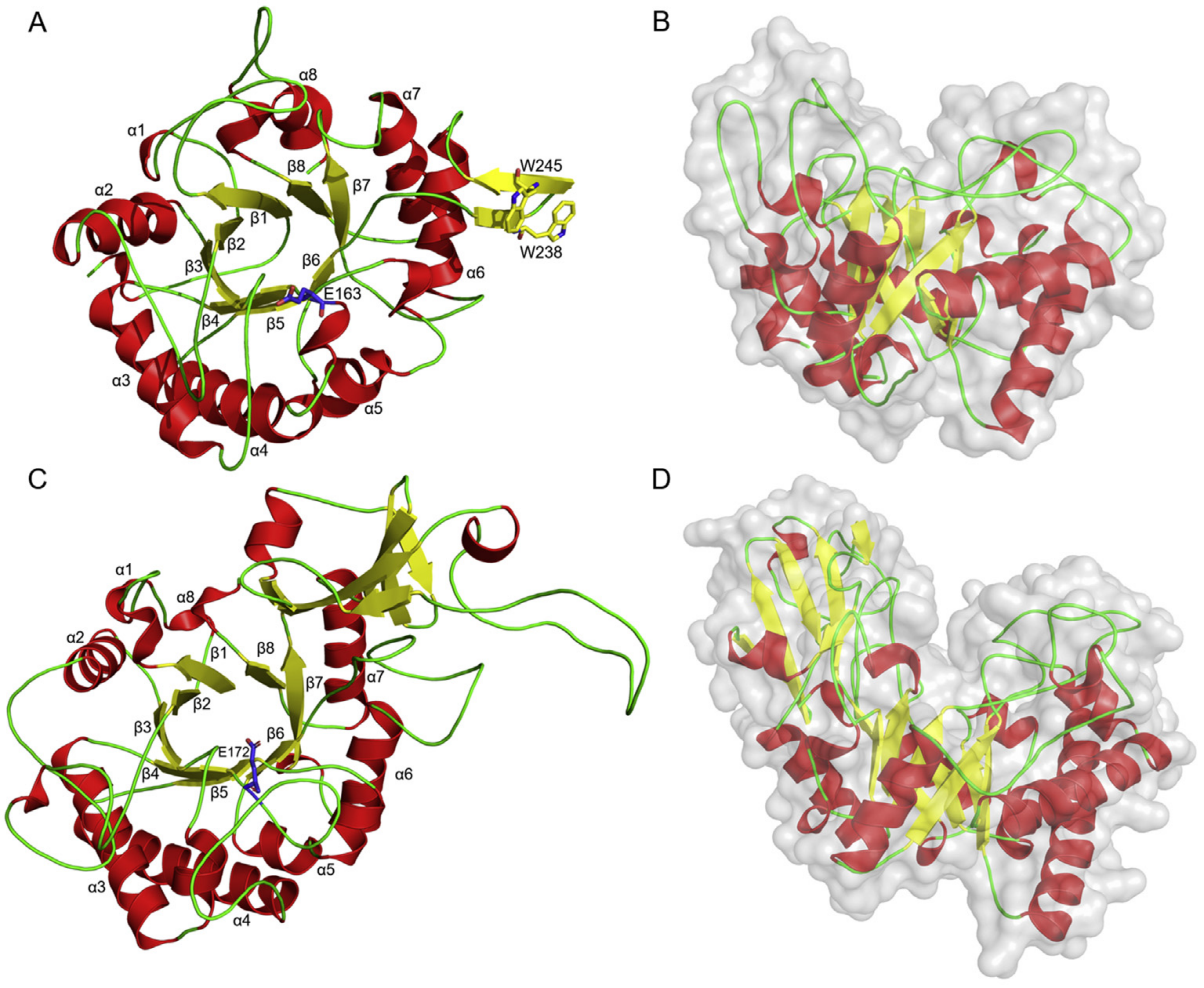
The overall 3D structural models (top view) of LmChiA and LmChiB catalytic domains with an (α/β)_8_ TIM barrel fold shown in ribbon drawings (A and C). The catalytic glutamates (E163 in LmChiA, and E172 in LmChiB) are positioned between β-strand 4 and α-helix 4 as shown by blue sticks. Two exposed tryptophan residues (W238 and W245) involved in substrate binding are shown by yellow sticks. Molecular surfaces with ribbon representation of LmChiA and LmChiB catalytic domains (side view) are shown in B and D. An α-helix is represented by red, β-strand by yellow, and loop by green.

**Figure 8:**
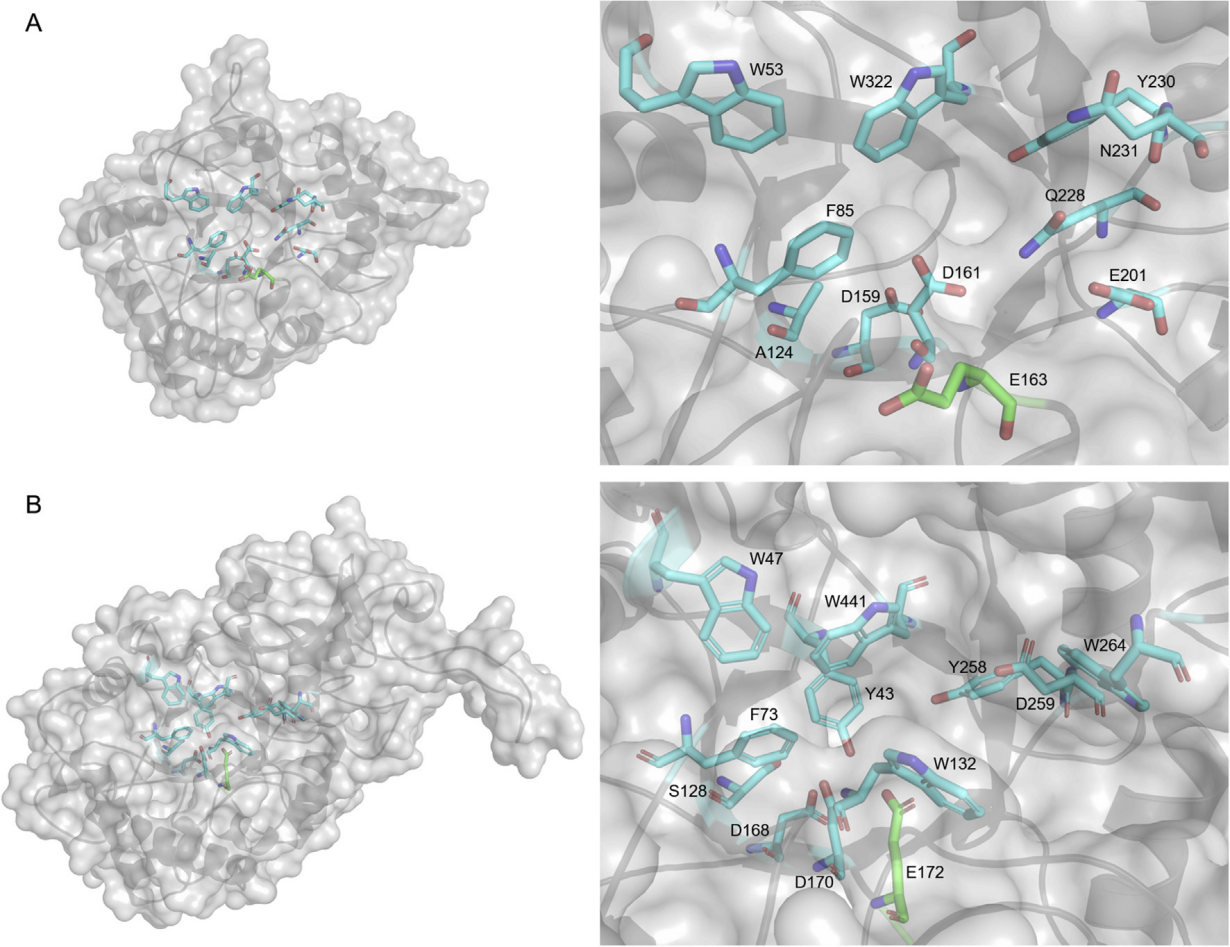
The active sites of LmChiA (A) and LmChiB (B) are formed with the key residues shown in the cyan stick representations. The crucial catalytic glutamate is represented as green stick. These amino acid residues are aligned on the (α/β)_8_ TIM barrel fold of catalytic region.

The 3D-models of the LmChiA and LmChiB CatDs were generated to investigate their structure-function relationships. Amino acid sequence alignments showed that the LmChiA catalytic domain shared highest sequence identity (63.21%) with that of SmChiC from *S. marcescens* (PDB code: 4AXN) (Payne et al., 2012), whereas the LmChiB catalytic domain shared highest sequence identity (32.59%) with that of BcChiA1 *B. circulans* (PDB code: 1ITX) (Matsumoto et al., 1999). Therefore, 4AXN and 1ITX were selected as suitable templates for LmChiA and LmChiB catalytic domain homology model building. The structural model of each chitinase was built by SWISS-MODEL and superimposed on their template structures to minimize the number and position of insertions and deletions. The structures of LmChiA and LmChiB catalytic domains are composed of eight β-strands and eight α-helices forming a common structure of (α/β)_8_ TIM barrel fold (Figure 7A-B). Interestingly, LmChiB has the chitin insertion domain (CID) on the top of the TIM barrel that gives it a close-deep cleft for substrate binding and is proposed to be important for processive hydrolysis of exochitinases (Figure 7C-D). It was difficult to predict the structure of CID since its sequence in LmChiB was not highly conserved with that of template BcChiA1 (30.76% sequence identity of only CID). On the other hand, the CID model was absent in the CatD of LmChiA between β-strands 7 and 8 of the TIM barrel fold. The active site of LmChiA CatD contained two tryptophan residues (Trp53 and Trp322) which are conserved in CatD of SmChiC (Trp238 and Trp245) (Figure 8). These residues are positioned in a small β-hairpin subdomain (residues 235–248). It was predicted that two exposed tryptophan residues might be involved in substrate binding and possibly serves as an attachment point to the chitin surface (Payne et al., 2012).

**Figure 9:**
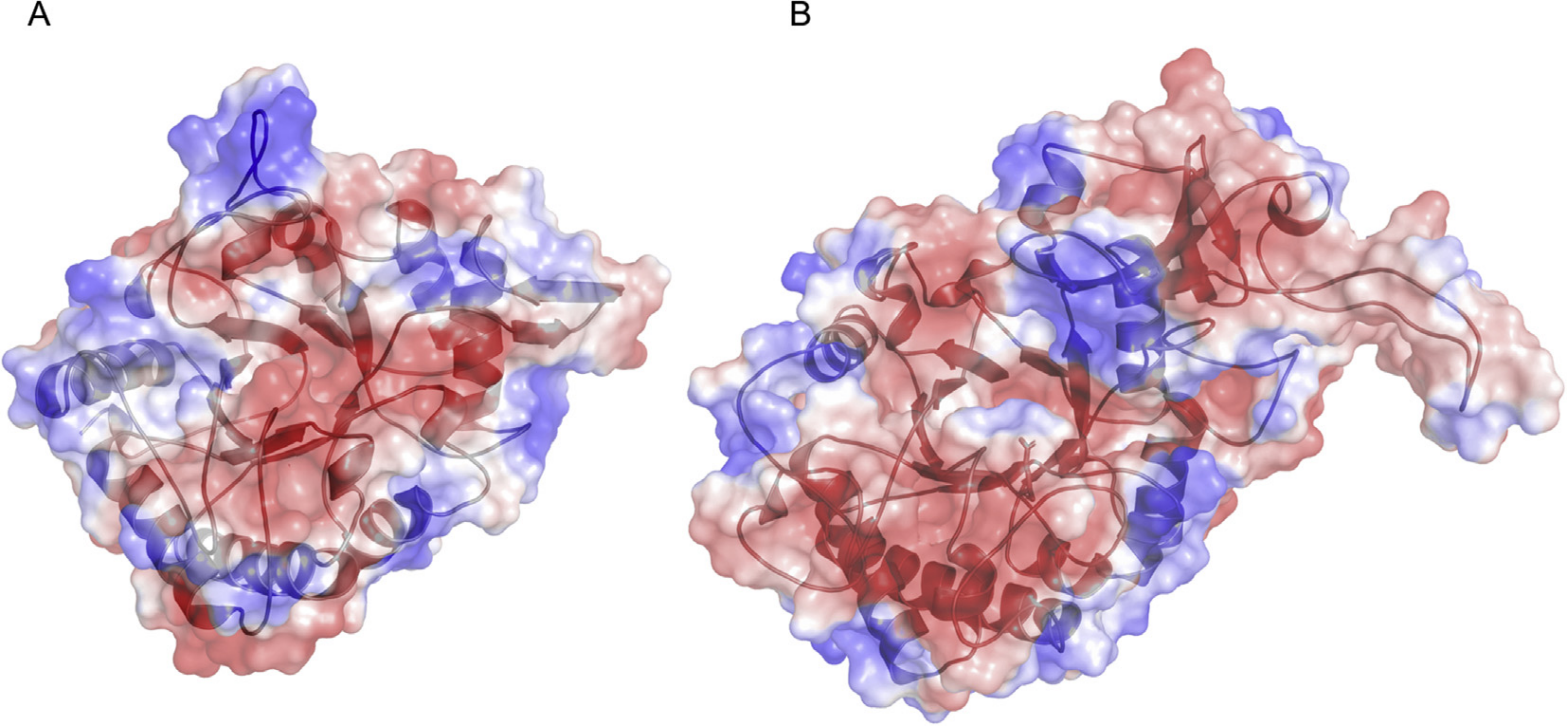
The electrostatic surfaces of LmChiA and LmChiB catalytic domains (top view) represent a negatively charged substrate-binding cleft. Electrostatic surfaces are colour-coded by charge (red is negative charge, blue is positive charge and white is hydrophobic).

The electrostatic surface potential of each model was calculated by PyMOL. Both chitinases contained negatively charged substrate-binding clefts suitable for the access and binding of chitin substrate (Figure 9). The cleft of LmChiA seemed to be more negative due to the presence of negatively charge amino acids in the DXDXE motif (Asp159, Asp161, Glu163) as well as Glu201. The crucial catalytic glutamate essential for substrate-assisted catalysis of LmChiA (Glu163) and LmChiB (Glu172) were located between β-strand 4 and α-helix 4 (Figure 8). The key residues involved in substrate recognition and catalytic mechanisms in the active sites of SmChiC, LlChi18A, and BcChiA, are completely conserved in LmChiA including Trp53, Phe85, Ala128, Asp159, Asp161, Glu163, Gln228, Tyr230, Asn231 and Trp322 (Hsieh et al., 2010; Vaaje-Kolstad et al., 2009) (Figure 8). For LmChiB, the aromatic residues within the deep substrate-binding cleft (Trp47, Trp132, Tyr258 Trp264 and Trp441) were highly conserved in BcChiA1 (Trp53, Trp164, Tyr279, Trp285 and Trp433) (Watanabe et al., 2003).

### 3.7 Construction of mutant chitinases and their enzymatic activity

Three conserved amino acid residues in DXDXE motif residing in the putative catalytic site of LmChiA and LmChiB were selected for site-directed mutagenesis to verify their significance in the chitinase activity of both chitinases. The mutant plasmids harboring a single amino acid substitution in *chiA* gene at position Asp159, Asp161 and Glu163, and *chiB* gene at position Asp168, Asp170 and Glu172 by alanine were designated as pChiA-D159A, pChiA-D161A, pChiA-E163A, pChiB-D168A, pChiB-D170A and pChiB-E172A, respectively. Ala was chosen because it is an electrically uncharged hydrophobic amino acid with less steric hindrance and used to identify the potential activity-enhancing mutations (Ni et al., 2015).

The enzymatic hydrolysis on colloidal chitin substrate was carried out in order to investigate the effect of the mutations on chitinolytic activity. All mutants decreased their activities on colloidal chitin substrate significantly in terms of both specific activity and relative activity (Table 5). Mutation of Asp159, Asp161 and Glu163 in LmChiA resulted in approximately 3-fold decrease in activity (30–32% of wild-type activity) whereas the activity of D168A, D170A and E172A of LmChiB mutants were greatly reduced (approximately 6-fold, 13–18% of wild-type activity). These results suggested that conserved Asp and Glu residues in DXDXE motif play an important role in the catalytic mechanism of LmChiA and LmChiB (Synstad et al., 2004; Vaaje-Kolstad et al., 2013).

**Table 5:** Specific activity of mutant and wild-type chitinase measured on colloidal chitin substrate.

Enzyme	Specific activity (U/μmol)	Relative specific activity (%)
Wild-type ChiA	2.01 ± 0.08	100
ChiA-D159A	0.66 ± 0.13	32.84
ChiA-D161A	0.75 ± 0.12	37.31
ChiA-E163A	0.62 ± 0.19	30.85
Wild type ChiB	11.61 ± 0.21	100
ChiB-D168A	1.51 ± 0.14	13.01
ChiB-D170A	2.20 ± 0.16	18.95
ChiB-E172A	1.56 ± 0.22	13.44

## 4 Discussion

A wide range of microorganisms, particularly bacteria, are considered as major sources of chitinase (Hamid et al., 2013). *L. monocytogenes* is considered to be a chitinolytic bacteria that produces two GH18 chitinases, LmChiA and LmChiB. GH18 chitinases have catalytic domains with an (α/β)_8_ TIM barrel fold and hydrolyze chitin through substrate-assisted catalysis. The conserved aromatic residues (Trp53, Phe85, Tyr230 and Trp322 in LmChiA, and Tyr43, Phe73, Trp132, Tyr258 and Trp441 in LmChiB) of LmChiA, LmChiB and other well-studied GH18 chitinases are identified as key aromatic residues that play important roles in substrate binding and chitin stabilization during the catalytic mechanism through hydrophobic stacking interactions (van Aalten et al., 2001; Vaaje-Kolstad et al., 2009; Watanabe et al., 2003). Family 18 chitinases can be classified into subfamilies A and B due to their similar yet distinct amino acid sequences. The major structural difference between subfamilies A and B is a small α+β fold CID, that is located at the top of the TIM barrel fold of the CatD in subfamily A creating a deep substrate-binding cleft, whereas subfamily B does not contain this CID. Based on structural characteristics, chitinases in subfamily A hydrolyze substrate with exo-activity, while subfamily B chitinases exhibit endo-activity (Watanabe et al., 2003).

From structure-based sequence analyses, the CID was found in the CatD sequence of LmChiB, and this CatD was closely related to that of BcChiA1 from *B. circulans* WL-12 (32.6% identity between the two CatD sequences). Both BcChiA1 and LmChiB have closed, deep-substrate binding grooves with highly conserved aromatic residues, with the CID located in the CatD, suggesting their action as exochitinase (Matsumoto et al., 1999; Vaaje-Kolstad et al., 2009). It has been proven that the CID of subfamily A chitinase BcChiA1 from *B. circulans* is required for the binding of substrates, the formation of hydrophobic cores and the orientation of enzyme to longer substrates (Matsumoto et al., 1999). The CatD of LmChiA possesses an open-shallow binding cleft and does not contain the CID. It is thus similar to that of SmChiC and can be grouped with subfamily B chitinases (Payne et al., 2012).

Additionally, the binding cleft aligned with highly conserved aromatic residues was also observed in LmChiB. These aromatic residues in the cleft are proposed to facilitate substrate binding and may have a role in sliding chitin substrate through the cleft (Hsieh et al., 2010). It has been shown that two conserved tryptophan residues (Trp164 and Trp285) in BcChiA1, are very important for processive hydrolysis, especially for crystalline-chitin substrates. Mutation of these residues (W164A and W285A) decreases activity against both crystalline and colloidal chitin (Watanabe et al., 2003). Furthermore, Trp97 and Trp220 in the CatD of SmChiB are involved in processivity and in enhancing the efficient degradation of insoluble chitin (van Aalten et al., 2001; Honda et al., 2016). These two tryptophan residues are also conserved in LmChiB (Trp132 and Trp264), indicating its processive exochitinase activity on insoluble chitin. The chitinolytic activity of LmChiB toward insoluble colloidal chitin was 2-fold greater than that of LmChiA. This result is consistent with the function of its conserved aromatic residues.

A FnIIID is found in LmChiB with conserved aromatic residues involved in forming hydrophobic cores. This domain functions as a domain linker and might be essential for the degradation of insoluble and crystalline chitins (Tomme et al., 1995). The ChBD of LmChiB is a member of the family 5/12 carbohydrate-binding module, containing conserved aromatic and hydrophobic residues. These residues are thought to form the core region of ChBD of BcChiA1 and to be essential for its chitin binding ability (Jee et al., 2002). However, their roles in hydrolysis of chitin have not been well elucidated. Possible functions of ChBD are to facilitate correct positioning of CatD, to contribute to processive action, and to promote local decrystallization of chitin substrate. Previous studies found that the presence of ChBD domains increases both substrate affinity and catalytic efficiency, especially for the crystalline chitins (Hashimoto et al., 2000; Yan and Fong, 2018).

The recombinants LmChiA and LmChiB from *L. monocytogenes* 10403S were effectively produced from *E. coli* with molecular masses of 35 and 78 kDa, respectively. Both chitinases were stable over a wide range of pH and temperature but exhibited some different enzymatic properties including kinetic parameters, pH optimum and substrate specificity. They showed catalytic activity toward natural substrate, colloidal chitin, and artificial substrates including [4NP-(GlcNAc)_2_] and [4NP-(GlcNAc)_3_], but exhibited no activity towards the [4NP-(GlcNAc)]. This finding suggested that LmChiA and LmChiB showed both exochitinase (chitobiosidase) and endochitinase activity toward 4-nitrophenyl substrates. These results are consistent with the hydrolytic activity of LmChiA from *L. monocytogenes* and StChiA from a bacterial pathogen, *Salmonella enterica* Typhimurium, that showed activity against only [4NP-(GlcNAc)_2_] and [4NP-(GlcNAc)_3_] (Leisner et al., 2009; Larsen et al., 2011). The analyses of degradation products from oligomeric substrate by HPLC have shown comparable results with 4-nitrophenyl hydrolysis. The hydrolysis products of both enzymes are mainly dimers with a smaller amount of monomer indicating their exochitinase (chitobiosidase) activity. Both chitinases hydrolyzed GlcNAc_6_ to produce trimers indicating their endo-chitinolytic activity. Although chitinases from *L. monocytogenes* (LmChiA and LmChiB) and *L. innocua* (LinChi35 and LinChi78) shared high sequence similarity, their hydrolysis mechanisms were different. LinChi78 and LinChi35 hydrolyzed oligomeric substrates in a processive exo- and nonprocessive endo-manner, respectively, and showed different reactivity toward oligomeric substrates (Honda et al., 2016). LmChiA and LmChiB showed the same reactivity toward oligomeric substrates. The difference in their catalytic activities might contribute to their distinct enzymatic properties such as pH dependence and *K*_m_ values.

The kinetic parameters toward [4NP-(GlcNAc)_2_], which is the best substrate for LmChiA and LmChiB, were also determined. The *K*_m_ values for both chitinases were higher than those for related GH18 chitinases including chitinases from *L. innocua* and *S. marcescens,* while their *k*_cat_ values were comparable to those of chitinases from *L. innocua* (Honda et al., 2016). Leisner et al. (2009) reported a *K*_m_ of 1600 μM for [4NP-(GlcNAc)_2_] hydrolysis by LmChiA which is comparable to 1622 μM in the present study, however the *k*_cat_ (33.57 s^−1^ in this study) was slightly different from that previously reported (21.6 s^−1^). This might have resulted from the use of different protein standards for the Bradford protein determination. LmChiB had an approximately 4-fold lower *K*_m_ and 2-fold higher *k*_cat_ than LmChiA, suggesting that the catalytic efficiency of LmChiB was greater than that of LmChiA. The optimal pH of LmChiA was determined to be pH 6.0, similar to that of SmChiC which also showed an acidic pH optimum. LmChiA contained an asparagine residue at position 231 which is conserved in SmChiC. This asparagine residue is proposed to be characteristic of GH18 chitinases with acidic pH optimum (Honda et al., 2016; Leisner et al., 2009; Synstad et al., 2004). In addition, LmChiA from *L. monocytogenes* was associated with infection of macrophages which contain acidic vacuolar compartments, indicating a role in the pathogenicity of LmChiA in such acidic pH host cells (Chatterjee et al., 2006).

Structure-based sequence alignments revealed that both chitinases contained a signature DXDXE motif. The conserved catalytic glutamate (Glu163 in LmChiA and Glu172 in LmChiB) in the DXDXE motif has been demonstrated to act as a crucial proton donor in the glycosidic bond cleavage. Furthermore, the two aspartates in the DXDXE motif (Asp159 and Asp161 in LmChiA, and Asp168 and Asp170 in LmChiB) were also shown to function in hydrolytic processing. The aspartate nearest the catalytic glutamate aids in the charge stabilization of the oxazolinium ion intermediate and facilitates the nucleophilic attack on the anomeric carbon during its interaction with the *N*-acetyl group of GlcNAc, whereas the second aspartate stabilizes the protonation of the first aspartate (Vaaje-Kolstad et al., 2013). The single mutation of Asp140, Asp142 and Glu144 in catalytic center of SmChiB from *S. marcescens* dramatically decreased its activity against (GlcNAc)_3_ analogue 4-methylumbelliferyl-β-D-*N*-*N′*-diacetylchitobioside [4-MU-(GlcNAc)_2_] substrate (Synstad et al., 2004). In this study, replacement of Asp159, Asp161 and Glu163 by Ala in LmChiA and Asp168, Asp170 and Glu172 by Ala in LmChiB significantly reduced the chitinolytic activity toward colloidal chitin substrate, indicating the significance of Asp and Glu residues in substrate hydrolysis. LmChiA has another essential glutamate (Glu201) that is conserved similar to Glu190 of BcChiA from *B. cereus*. This glutamate might be involved in the regulation of product release and its mutant (E190Q) decreases the hydrolytic activity (Hsieh et al., 2010). LmChiB also contains the SXGG motif followed by a tryptophan residue which is important for the hydrolysis of insoluble chitin by processive chitinases. In the LmChiA sequence, a serine residue in the SXGG motif is substituted with an alanine residue (Ala124). This serine in GH18 chitinases is responsible for the negative charge stabilization on the first aspartate of the DXDXE motif during catalytic reactions. However, it was demonstrated that this charge stabilization of SmChiB from *S. marcescens* could be accomplished by a tyrosine residue, in addition to serine, in the SXGG motif (Synstad et al., 2004). Even if the LmChiA catalytic region lacks serine, it still contains a tyrosine residue (Tyr49, corresponding to Tyr10 in SmChiB). The CatD of LlChi18A from *Lactococcus lactis* also lacks this serine, and the conserved CatDs found in other family18 chitinases have substitutions at either the serine or tyrosine suggesting that family18 chitinases are resistant to substitutions of either of these two residues (Vaaje-Kolstad et al., 2009).

Generally, functional chitinolytic systems consist of both exochitinase and endochitinases which act synergistically on chitin substrates, leading to increases in substrate accessibility and catalytic efficiency (Paspaliari et al., 2015). Thus, LmChiA and LmChiB are thought to be part of the chitinolytic machinery of *L. monocytogenes* 10403S which convert natural biopolymers into nutrient sources. These chitinases might have potential in the utilization of chitin waste and other biotechnological applications.

## Declarations

### Author contribution statement

Wasinee Churklam: Performed the experiments; Wrote the paper.

Ratchaneewan Aunpad: Conceived and designed the experiments; Analyzed and interpreted the data; Wrote the paper.

### Funding statement

Miss Wasinee Churklam was supported by 10.13039/501100004396Thailand Research Fund under the Royal Golden Jubilee Ph.D. programme (PHD/0159/2556,5.L.TU/55/I.1). This work was partially supported by Research Unit in Antimicrobial Agent and Application, Thammasat University.

### Competing interest statement

The authors declare no conflict of interest.

### Additional information

No additional information is available for this paper.

## Appendix A Supplementary data

Supplementary file 1.docx
