# Supplementary material for: Enzymatic characterization and structure-function relationship of two chitinases, LmChiA and LmChiB, from Listeria monocytogenes
Source: Heliyon. 2020 Jul 1;6(7):e04252. doi: 10.1016/j.heliyon.2020.e04252 (PMC7334433; doi:10.1016/j.heliyon.2020.e04252)

**Supplementary File 1**

**Supplementary File 1:** SDS-PAGE analysis of LmChiA (1) and LmChiB (2) during purification steps.


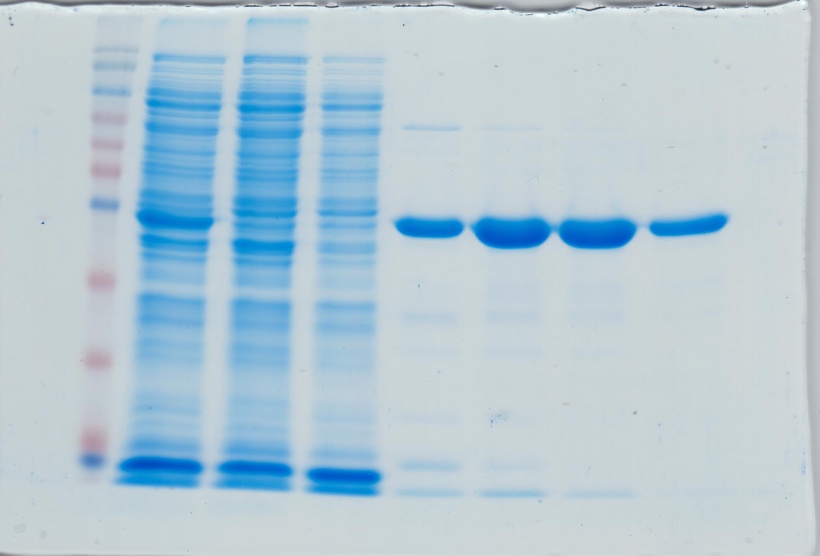
 (1)

(2)


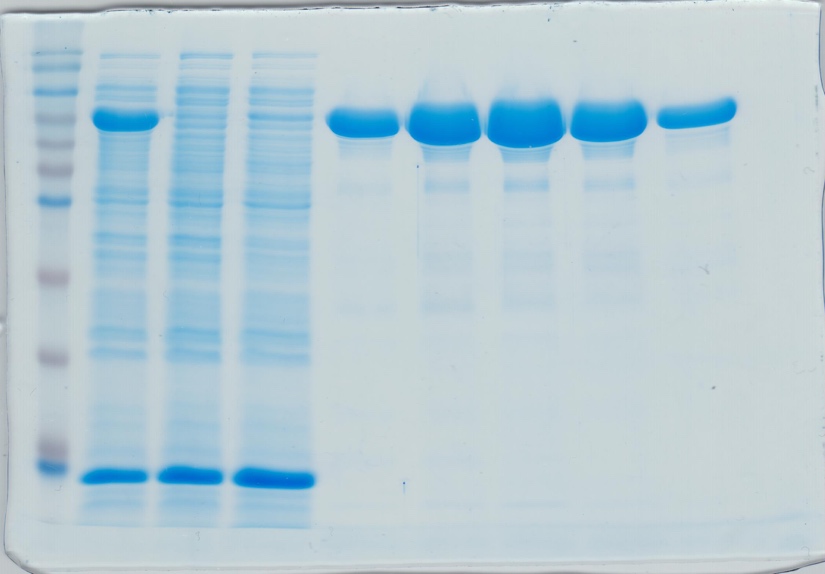

Supplement: Supplementary file 1 — docx [file mmc1.docx]
